# The TGFβ-SMAD3 pathway inhibits IL-1α induced interactions between human pancreatic stellate cells and pancreatic carcinoma cells and restricts cancer cell migration

**DOI:** 10.1186/s13046-016-0400-5

**Published:** 2016-07-29

**Authors:** Vegard Tjomsland, Dagny Sandnes, Ewa Pomianowska, Smiljana Torbica Cizmovic, Monica Aasrum, Ingvild Johnsen Brusevold, Thoralf Christoffersen, Ivar P. Gladhaug

**Affiliations:** 1 Department of Hepato-pancreato-biliary Surgery, Institute of Clinical Medicine, University of Oslo, Oslo, Norway; 2 Department of Pharmacology, Institute of Clinical Medicine, University of Oslo, Oslo, Norway; 3 Department of Hepato-pancreato-biliary Surgery, Oslo University Hospital, Rikshospitalet, Oslo, Norway; 4 Department of Oral Biology, University of Oslo, Oslo, Norway; 5 Department of Pediatric Dentistry and Behavioral Science, Faculty of Dentistry, University of Oslo, Oslo, Norway

**Keywords:** Pancreatic adenocarcinoma, Tumor stroma, Pancreatic stellate cells, TGFβ, IL-1α

## Abstract

**Background:**

The most abundant cells in the extensive desmoplastic stroma of pancreatic adenocarcinomas are the pancreatic stellate cells, which interact with the carcinoma cells and strongly influence the progression of the cancer. Tumor stroma interactions induced by IL-1α/IL-1R1 signaling have been shown to be involved in pancreatic cancer cell migration. TGFβ and its receptors are overexpressed in pancreatic adenocarcinomas. We aimed at exploring TGFβ and IL-1α signaling and cross-talk in the stellate cell cancer cell interactions regulating pancreatic adenocarcinoma cell migration.

**Methods:**

Human pancreatic stellate cells were isolated from surgically resected pancreatic adenocarcinomas and cultured in the presence of TGFβ or pancreatic adenocarcinoma cell lines. The effects of TGFβ were blocked by inhibitors or amplified by silencing the endogenous inhibitor of SMAD signaling, SMAD7. Pancreatic stellate cell responses to IL-1α or to IL-1α-expressing pancreatic adenocarcinoma cells (BxPC-3) were characterized by their ability to stimulate migration of cancer cells in a 2D migration model.

**Results:**

In pancreatic stellate cells, IL-1R1 expression was found to be down-regulated by TGFβ and blocking of TGFβ signaling re-established the expression. Endogenous inhibition of TGFβ signaling by SMAD7 was found to correlate with the levels of IL-1R1, indicating a regulatory role of SMAD7 in IL-1R1 expression. Pancreatic stellate cells cultured in the presence of IL-1α or in co-cultures with BxPC-3 cells enhanced the migration of cancer cells. This effect was blocked after treatment of the pancreatic stellate cells with TGFβ. Silencing of stellate cell expression of SMAD7 was found to suppress the levels of IL-1R1 and reduce the stimulatory effects of IL-1α, thus inhibiting the capacity of pancreatic stellate cells to induce cancer cell migration.

**Conclusions:**

TGFβ signaling suppressed IL-1α mediated pancreatic stellate cell induced carcinoma cell migration. Depletion of SMAD7 upregulated the effects of TGFβ and reduced the expression of IL-1R1, leading to inhibition of IL-1α induced stellate cell enhancement of carcinoma cell migration. SMAD7 might represent a target for inhibition of IL-1α induced tumor stroma interactions.

## Background

Pancreatic ductal carcinoma (PDAC) is a highly lethal disease, which usually has metastasized before diagnosis [1]. Surgical resection, the only potentially curative treatment, is only possible in 15–20 % of the patients [1, 2]. The disease is also largely resistant to chemotherapy and radiotherapy [1]. Despite important advances in the understanding of the pathobiology of this cancer over the last few decades [3], no real improvements in the clinical outcome have been achieved, and the 5-year survival rate is still less than 5 % [4, 5].

Many lines of evidence have demonstrated the crucial role of the microenvironment of tumors [6]. Malignant cells interact with a variety of stromal cell types and extracellular matrix components, in complex manners closely related to inflammatory processes [7]. These interactions are mediated by a number of growth factors, cytokines, and other locally active molecules, which exert fundamental influences on the properties of the cancer, such as its growth rate and propensity to invade and metastasize [7, 8]. In pancreatic tumors, a characteristic feature is the extensive desmoplastic reaction consisting of extracellular matrix (ECM) and stromal cells, sometimes comprising up to 90 % of the total tumor mass [9]. It is now clear that the cells responsible for the desmoplastic reaction are a special type of cancer-associated fibroblasts, the pancreatic stellate cells (PSCs) [8, 10]. In the normal pancreas, they are quiescent, lipid-storing cells, however, in pancreatic injury or stress, the PSCs can be activated by cytokines and other factors released from injured ductal epithelium and stromal cells and undergo transdifferentiation to myofibroblast-like cells [11]. These activated PSCs have proliferative capacity and ability to produce ECM and several tumor-promoting growth factors [10]. The PSCs have been strongly implicated in the progression of pancreatic cancer [12], and evidence suggests that they function in a reciprocal stimulatory interaction with the carcinoma cells through various active factors [13, 14]. However, this view has recently been challenged by experimental data from genetically engineered mice, showing that depletion of stromal components enhanced tumor growth in pancreatic carcinomas [15]. These discrepant results [9, 12–16], probably reflect an even more complex tumor-stroma relationship than has been recognized.

The interaction between pancreatic carcinoma and stellate cells largely takes place through various active factors. Two of these factors are interleukin 1α (IL-1α) and transforming growth factor beta (TGFβ) [17]. IL-1α is a highly proinflammatory cytokine, abundantly present in the tumor microenvironment, where it is released from various stromal cells as well as from carcinoma cells [18]. Acting via its receptor IL-1R1, which belongs to the interleukin-1 receptor/Toll-like receptor superfamily [19], IL-1α exerts multiple effects in the tumor stroma, several of which are tumor-promoting [20]. In pancreatic carcinoma, IL-1α was found to sustain the expression of inflammatory factors in the microenvironment and enhance the migratory capacity of the cancer cells [21]. Since mutation of *KRAS* is a crucial event in pancreatic carcinogenesis [22], it is of particular interest that studies in a mouse model have strongly suggested that IL-1α is a link between mutated, oncogenic Ras (*Kras*^*G12D*^) and the tumor-promoting inflammatory microenvironment required for the development of these cancers [23].

TGFβ exerts profound, pleiotropic, context-dependent regulations of normal and malignant cells [24–26]. Its many effects in normal physiology include inhibitory control of normal epithelial cell growth and regulation of the immune system [27, 28]. In malignancy, TGFβ has several and multifaceted roles. It exerts suppressive effects on tumor-promoting inflammation and on early stages of carcinogenesis, but, on the other hand, TGFβ is a major factor enhancing tumor progression, epithelial-mesenchymal transition (EMT), and invasiveness and metastatic capacity [24, 28, 29]. The canonical TGFβ signaling cascade involves binding and recruitment of cell surface kinase receptors (TβRII and TβRI) and intracellular activation of SMAD2 or SMAD3 proteins which form a complex with SMAD4 and subsequently translocate into the nucleus, interacting with other transcription factors to regulate the expression of target genes. The TGFβ/SMAD signaling cascade is regulated by endogenous inhibitors, SMAD6 and SMAD7 [24, 25]. Although TGFβ preferably signals via the SMAD pathway, it can also activate other pathways that collectively are referred to as non-canonical TGFβ signaling which complements the action of SMAD [26]. In pancreatic cancer, the effects of TGFβ are complex and not fully understood [30]. In particular, the role of TGFβ in signal cross-talk between carcinoma cells and pancreatic stellate cells is of interest for identification of targets for novel therapeutic strategies and warrants further study. In the present work we have studied effects of IL-1α and TGFβ in stromal cell-induced migration of pancreatic carcinoma cells. The data show that TGFβ signaling suppressed IL-1α-mediated stellate cell-induced carcinoma cell migration, indicating that TGFβ inhibits tumor promoting effects of human pancreatic stellate cells.

## Methods

### Patients

The study protocol and patient consent documents were approved by the Regional Committee for Medical and Health Research Ethics (REC South East, project number 2010/694a), and was in compliance with the Helsinki Declaration. Written informed consent was obtained from all study participants. The study included only adults.

### Cells, isolation and culture

Human pancreatic stellate cells (PSCs) were isolated from pancreatic tumor tissue obtained during pancreatic surgery from patients with resectable pancreatic head adenocarcinoma and cultured by the outgrowth method developed by Bachem et al. [31] as explained elsewhere [32]. The purity of the PSCs was assessed by morphology and cytofilament staining of α-SMA and vimentin. None of the cells were positive for CK7 or CK20. All experiments were performed using cell populations between passage 4 and 8. The primary PDAC cell line PC013 was propagated from PDAC tumor tissue biopsies as described elsewhere [21]. BxPC-3 and CAPAN2 were purchased from ATCC (Manassas, VA, USA). All cell lines were cultured in Dulbecco’s modified Eagle’s medium containing 4.5 g/l glucose (DMEM). The media were supplemented with 100 μg/ml Pen-Strep, Glutamax and 10 % fetal bovine serum (FBS) (Life Technologies). For IL-1α (Biolegend, Sandiego, CA), IL-1RA (Kineret® (Anakinra) a gift from Swedish Orphan Biovitrum AS, Norway), TGFβ and PDGF (R&D Systems Europe, Abingdon, UK) stimulation, the PSCs were cultured to confluence, washed with NaCl and cultured in serum free (SF) DMEM medium supplemented with 1 ng/ml IL-1α, and/or 2 ng/ml TGFβ, 10 μg/ml IL-1Ra or 10 ng/ml PDGF. Supernatants were harvested after 4 days of culture, centrifuged and stored at −30 °C until use. TGFβ signaling in PSCs were blocked by the ALK-5 inhibitor A-83-01 (5 μM) (R&D Systems) or the SMAD3 inhibitor SIS3 (Sigma-Aldrich, Oslo, Norway) (5 μM) in the presence of 2 ng/ml TGFβ or conditioned medium from BxPC-3, CAPAN2 or PC013 cells cultured in serum free conditions for 3 days. All gene expression experiments were conducted for 72 h before lysing the cells, while PSC supernatants were harvested after 4 days of culture and stored in −30 °C until use.

### Gene silencing of SMAD7 by siRNA

Expression of SMAD7 was silenced using SMARTpool: ON-TARGETplus SMAD7 siRNA (GE Dharmacon, Lafayette, CO) and non-silencing siRNA was used as control (GE Dharmacon) according to the manufacturer's protocol. Final concentration of siRNA was 100 nM in 2 ml DMEM medium supplemented with 1 % FBS. The cells were transfected with siRNA for 72 h, washed and incubated with SF medium for 48 h before adding 1 ng/ml IL-1α. The PSCs were incubated for another 72 h and PSC supernatants were harvested (5 days after removing siRNA from the PSCs). The PSCs were lysed for protein and RNA experiments and stored in −30 °C until use.

For the co-culture assays, 5×10^5^ BxPC-3 cells were seeded per Transwell® insert (pore size, 0,4 μm) (Corning Incorporated, Corning, NY) and cultured for 24 h before placing the inserts into 6-well plates containing a confluent layer of either untreated, control siRNA or SMAD7 gene silenced PSCs. Cancer cells and PSCs were co-cultured for 12 h before removing the insert. The PSCs were cultured for further 60 h and PSC supernatants were harvested after a total of 5 days after removing the siRNA. The PSCs were lysed for protein and RNA experiments and stored at −30 °C until use.

### RNA extraction and real-time quantitative RT-qPCR

Total RNA was prepared from the samples using RNA Easy Mini kit (Qiagen Inc, Valencia, CA) and cDNA was synthesized with SuperScript III Reverse Transcriptase First-Strand cDNA Synthesis kit according to the manufacturer’s protocol (Life Technologies, Carlsbad, CA). Quantitative PCR was performed with Platinum SYBR Green Master Mix (Life Technologies) on 7900 Real-Time PCR system with 7900 System SDS 2.3 Software (Life Technologies) according to the manufacturer’s protocol. Specific primers for SMAD2, SMAD3, SMAD7, IL-6, IL-8 and IL-1R1 (Life Technologies) were used. Glyceraldehyde-3-phosphate dehydrogenase (GAPDH) was utilized as housekeeping control gene. The primers were designed using Primer-BLAST [33]. All reactions were performed in triplicates including non-template controls. The results were analysed using the ΔΔCt method [34]. The relative gene expression raw data were normalized to GAPDH and presented as relative gene expression for each gene.

### ELISA analysis

The levels of IL-1α and TGFβ in supernatants from PC013, BxPC-3, CAPAN2 cells and PSCs were assessed after culturing the cells for 3 days in DMEM medium supplemented with 1 % FBS. The supernatants were harvested and the concentration of IL-1α (Biolegend, San Diego, CA) and TGFβ (Nordic BioSite, Oslo, Norway) were measured by ELISA according to the manufacturers’ protocol.

### Immunocytochemistry and immunohistochemistry

Cultured PSCs were immunostained with anti-human IL-1R1 antibodies (ab59995, ABcam, Cambridge, UK), visualized by Alkaline Phosphatase Red (Biocare Medical, Concord, CA) and DAPI (Jackson ImmunoResearch, West Grove, PA) as described elsewhere [21]. Representative PDAC tissue was stained for IL-1R1 (04–465, Merck Millipore, Darmstadt, Germany) as described previously [21]. Positive cells were detected with ImmPRESS™ HRP Polymer Detection Kit (Vector laboratories, Peterborough, UK).

### Western blot analysis

Total cell lysates from PSCs grown to confluence in 12 well plates in the presence of either control medium, TGFβ (2 ng/ml) and/or 10 μM SIS3, were prepared in Laemmli buffer and electrophoresed on 12 % (w/v) polyacrylamide gels (acrylamide: N’N’-bis-methylene acrylamide 30:1). This was followed by protein electrotransfer to nitrocellulose membranes and immunoblotting over night with antibodies against, IL-1R1 (04–465, Merck Millipore, Darmstadt, Germany), SMAD7 (MAB2029, R&D Systems) and GAPDH (2118, Cell Signaling Technology, Boston, MA), respectively. Immunoreactive bands were visualized using HRP conjugated secondary antibodies (LI-COR, Lincoln, NE) and LumiGLO HRP Chemiluminescent Substrate (KPL Protein research Products, Gaithersburg, MD). Images were acquired using EpiChemi II Darkroom (UVP, Upland, CA) and band intensity processed using FIJI software as described by Schindelin et al. [35].

### Migration assay

Cell migration was assessed using a scratch assay [36]. 2×10^5^ BxPC-3 cells in 100 μl DMEM medium supplemented with 10 % FBS were seeded in 12 well culture plates pre-marked with three ink marks under the bottom of each well. The cells were left to adhere for 2 h; then, 1 ml of serum free medium (SF) was added, and the cells were incubated overnight to confluence. A scratch was made with a 100 μl pipette tip. The marks under the dishes served to ensure that exactly the same observation field was studied during the observation period. After scratching, the cells were washed twice with NaCl and then kept in SF medium or PSC supernatants for 24 h. The scratch wounds were observed in a Zeiss Axiovert 25 inverted microscope with a 5× objective (Carl Zeiss AS, Oslo, Norge). Images (each 1.4 × 1.0 mm), taken right after the addition of SF or PSC supernatants and at 24 h, were obtained with a Zeiss AxioCam ICc3 (Carl Zeiss AS). For each picture, the wound area was measured by FIJI software as described by Schindelin et al. [35]. Per cent wound closure was calculated for the time point of observation based on the mean of 2–3 observations from each scratch.

### Statistical analysis

The statistical analysis was performed with GraphPad Prism 5 (GraphPad Software), *p* < 0.05 was considered statistically significant and error bars throughout indicate standard error of the mean (SEM). Normalized data were analysed by paired t-test, multiple comparisons were analysed using ANOVA including Bonferroni correction and correlation determined by Pearson correlation coefficient.

## Results

### TGFβ down-regulates the expression of IL-1R1 in pancreatic stellate cells

**Fig. 1:**
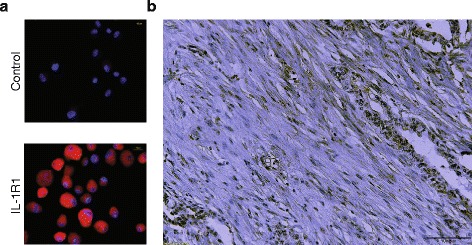
IL-1R1 expression in pancreatic stellate cells (PSCs) and PDAC tissue. **a** The expression and localization of IL-1R1 (*red*) were analysed by immunofluorescence in PSCs established from PDAC tumors. **b** Immunohistochemistry of IL-1R1 (*brown*) in PDAC tissue

**Fig. 2:**
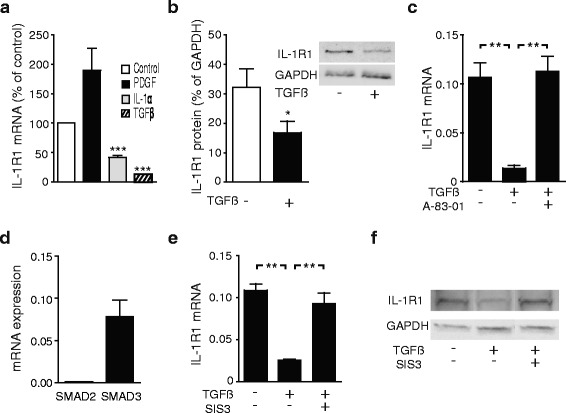
TGFβ down-regulates the expression of IL-1R1 in pancreatic stellate cells. **a** PSCs cultured in the presence of TGFβ, IL-1α or PDGF were analysed by qPCR and IL-1R1 gene expression normalized to unstimulated PSCs. **b** Protein expression was measured 3 days after exposure to TGFβ and analysed by western blotting. IL-1R1 was quantified by measuring the band intensity (*n* = 4). **c** IL-1R1 gene expression was analysed in PSCs incubated in the presence of TGFβ (2 ng/ml) and the TGFβ signaling inhibitor, A-83-01 (5 μM). **d** PSC expression of SMAD2 and SMAD3 mRNA was assessed by qPCR. **e** PSCs were stimulated for 3 days with TGFβ (2 ng/ml) in the presence or absence of the SMAD3 inhibitor, SIS3 (5 μM). The gene expression of IL-1R1 was quantified by qPCR and (**f**) protein expression visualized by western blotting. Error bars represent S.E.M.; *p < 0.05, ** *p* < 0.005, ***p < 0.001

Interleukin-1 receptor 1 (IL-1R1) is the exclusive receptor for IL-1, and its expression level on a cell is a determinant for signaling strength and biological response to IL-1 [37, 38]. High expression of IL-1R1 on cultured PSCs was demonstrated by immunofluorescence (Fig. 1a). Immunohistochemistry of intact PDAC tissue showed expression of IL-1R1 in both stroma cells and cancer cells (Fig. 1b). Several biological factors have been found to modulate the effects of IL-1 by down-regulating or enhancing the expression of IL-1R1 [39, 40]. We examined how incubation of PSCs in the presence of IL-1α, platelet-derived growth factor (PDGF), or TGFβ, influenced their expression of IL-1R1. Figure 2a shows that in long-term culture (3 days) of PSCs, a single addition of PDGF (10 ng/ml) increased the expression of the IL-1R1 gene at the mRNA level, while both IL-1α (1 ng/ml) and TGFβ (2 ng/ml) strongly decreased the expression. The protein level of IL-1R1 was also significantly decreased upon incubation with TGFβ as compared to untreated controls (Fig. 2b). Further support for a role of TGFβ in down-regulation of IL-1R1 in PSCs was obtained by the use of A-83-01, a blocker of TGFβ type I receptor ALK5 kinase activity, which inhibits SMAD2/3 activation. Thus, A-83-01 abolished the inhibitory effects of TGFβ on IL-1R1 mRNA expression in PSCs (Fig. 2c). To investigate the relative contributions of SMAD2 and SMAD3 we examined their expression in PSCs. As shown in Fig. 2d, PSCs expressed high levels of SMAD3 and virtually no SMAD2. To investigate the involvement of SMAD3 in the down-regulation of IL-1R1, we incubated PSCs in the presence of SIS3, a potent SMAD3 inhibitor, upon TGFβ exposure. SIS3 abrogated the inhibitory effects of TGFβ and kept the PSC expression of IL-1R1 close to the control level (Fig. 2e-f). Together, these results provide support for a role of the TGFβ-SMAD2/3 pathway in the down-regulation of IL-R1 expression in PSCs.

### TGFβ down-regulates IL-1α induced cytokine production in pancreatic stellate cells

**Fig. 3:**
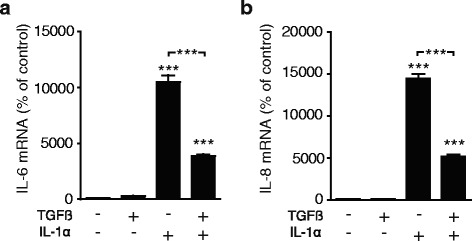
Pre-treatment with TGFβ inhibits IL-1α induced up-regulation of IL-6 and IL-8 in pancreatic stellate cells. The PSCs were incubated for 3 days in the presence or absence of 2 ng/ml recombinant TGFβ before adding 1 ng/ml IL-1α to the cells. The expression of (**a**) IL-6 and (**b**) IL-8 mRNA were measured by qPCR after 4 h of incubation. Error bars represent S.E.M.; ***p < 0.001

IL-1α stimulates cytokine production, including IL-6 and IL-8 in PSCs [21]. Since TGFβ inhibits IL-1R1 expression in PSCs, we next examined whether TGFβ affected IL-1α stimulation of PSC cytokine production. TGFβ significantly inhibited the effects of IL-1α on PSC expression of IL-6 (Fig. 3a) and IL-8 (Fig. 3b).

### SMAD7 is involved in the regulation of IL-1R1 expression in pancreatic stellate cells

**Fig. 4:**
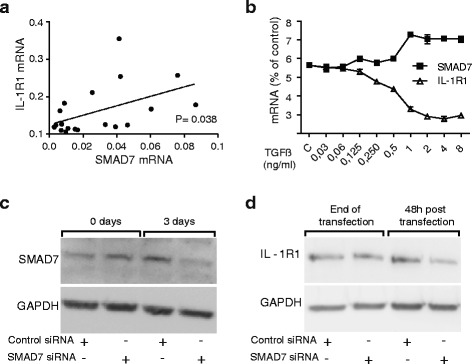
SMAD7 is involved in the regulation of IL-1R1 expression in PSCs. **a** Gene expression of SMAD7 and IL-1R1 from PSCs established from different patients (*n* = 19) were analysed by qPCR. PSCs were transfected with non-signaling siRNA (control) or SMAD7 siRNA (100nM) for 3 days. Correlation was determined using Pearson correlation coefficient. *p < 0.05. **b** Pancreatic stellate cells were incubated in the presence of increasing concentrations of recombinant TGFβ (30 pg/ml – 8 ng/ml) to assess the optimal concentration required to down-regulate the gene expression of IL-1R1 and to investigate the impact of TGFβ on PSC expression of SMAD7. **c** SMAD7 protein expression was visualized by western blotting after 3 days of culture and (**d**) IL-1R1 was visualized after additional 2 days in serum free medium

We measured the gene expression of SMAD7 and IL-1R1 in series of primary PSC cell preparations (*n* = 19) established in culture from PDAC patients, and found a significant (*P* = 0.038) correlation between SMAD7 and IL-1R1 gene expression (Fig. 4a). TGFβ increased the level of SMAD7 mRNA in the PSCs (Fig. 4b). Furthermore, when the effects of TGFβ were tested over a range of concentrations (30 pg/ml – 8 ng/ml), the upregulation of SMAD7 expression and the down-regulation of IL-1R1 expression corresponded closely, appearing around 1 ng/ml and being maximal at ≥ 2 ng/ml (Fig. 4b), suggesting a potential role for SMAD7 in the regulation of IL-1R1 expression in PSCs. Finally, a relationship between SMAD7 and IL-R1 expression was further supported by experiments which showed that silencing of SMAD7 expression, using a SMAD7-specific siRNA (Fig. 4c), was associated with reduced protein expression of IL-1R1 compared to the controls (Fig. 4d). These results are consistent with a role of SMAD7 as an enhancer of IL-1 signaling in PSCs due to its attenuation of TGFβ.

### Pancreatic adenocarcinoma cells inhibit the expression of IL-1R1 on the stellate cells: Role of IL-1α and TGFβ

Further studies aimed at exploring some of the interactions between PDAC cells and PSCs, focusing on the roles of IL-1α and TGFβ. First we investigated the expression of IL-1α and TGFβ in conditioned medium from PDAC cell lines and from PSCs. BxPC-3 and PC013 produced IL-1α, while conditioned medium from CAPAN2 and PSCs was IL-1α negative. TGFβ was detected at similar high levels in the conditioned medium from all the PDAC cell lines. PSCs also produced TGFβ, but at lower levels compared to the cancer cell lines (Table 1).

**Table 1:** Production of IL-1α and TGFβ by PDAC cell lines and pancreatic stellate cells

Cell type	IL-1α pg/ml 10^5^ cells	TGFβ pg/ml 10^5^ cells
BxPC-3	20.6	7553
CAPAN2	neg. ^a^	7167
PC013	38.0	7448
PSC	neg. ^a^	4375

IL-1α and TGFβ levels were measured by ELISA in conditioned medium from pancreatic cancer cell lines (PC013, BxPC-3, CAPAN2) and PSCs after 3 days of culture. The results are presented in pg/ml/10^5^ cells

^a^Below the lower detection limit

**Fig. 5:**
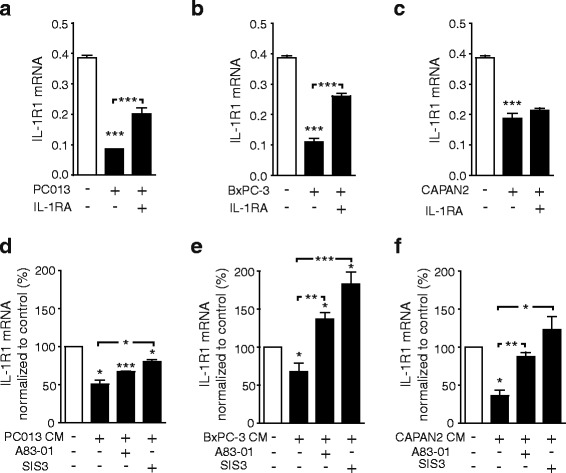
TGFβ inhibitors reduce the inhibitory effects of cancer cells on PSCs expression of IL-1R1. **a**-**c** The pancreatic cancer cell lines; (**a**) PC013, (**b**) BxPC-3 and (**c**) CAPAN2 were co cultured with PSCs in the presence of IL-1 receptor antagonist (IL-1RA) (10 µg/ml). **d**-**f** PSCs were cultured for 3 days in serum free conditioned medium (CM) from (**c**) PC013, (**e**) BxPC-3 and (**f**) CAPAN2 cell lines and TGFβ signaling inhibited by A-83-01 (5 μM) and SIS3 (5 μM). The IL-1R1 gene expression was analysed in PSCs by qPCR and the result shown as relative expression normalized to GAPDH or unstimulated controls. Error bars represent S.E.M.; *p < 0.05, **p < 0.005, ***p < 0.001

To investigate effects exerted by PDAC cells on IL-1R1 expression in PSCs, we co-cultured PSCs and the PDAC cell lines PC013, BxPC-3, and CAPAN2. The presence of either of these PDAC cell lines significantly reduced PSC expression of IL-1R1 (Fig. 5a-c suggesting possible involvement of paracrine signaling mechanisms acting between the cancer cells and the PSCs. To examine the role of IL-1α produced by the cancer cell lines, the co-cultures were incubated in the presence of anakinra, a recombinant, non-glycosylated form of the human interleukin-1 receptor antagonist (IL-1RA). This increased the expression of IL-1R1 in the PSCs and reduced the inhibitory effects exerted by the IL-1α positive cancer cell lines PC013 and BxPC-3 [21] (Fig. 5a-b), while the expression of IL-1R1 in PSCs co-cultured with the PDAC cell line CAPAN2, which is IL-1 negative [41], was not affected by the presence of IL-1RA (Fig. 5c).

We next examined the possible involvement of TGFβ from the cancer cells in the observed PDAC cell-induced down-regulation of IL-1R1 expression in PSCs. To prevent any direct effects of the TGFβ signaling inhibitors (A-83-01 and SIS3) on the cancer cell lines, conditioned media from PC013, BxPC-3 or CAPAN2 cells were added to the PSCs and cultured in the presence or absence of these inhibitors as an alternative to co-culture. All these conditioned media suppressed IL-1R1 mRNA to varying degrees (Fig. 5d-f). Either A-83-01 or SIS3 partly counteracted the down-regulatory effect of PC013-conditioned medium on PSC expression of IL-1R1 (Fig. 5d) and completely abolished the inhibition exerted by the media obtained from CAPAN2 and BxPC-3 cells (Fig. 3e-f). It may be noted that in PSCs incubated with BxPC-3 conditioned medium, these TGFβ pathway inhibitors significantly increased the expression of IL-1R1 compared to unstimulated controls (Fig. 5e). Taken together, these results suggest that both IL-1α, through an autoinhibitory feedback, and TGFβ mediate PDAC cell-induced down-regulation of IL-1R1 expression in PSCs.

### TGFβ treatment of stellate cells reduces carcinoma cell migration by inhibiting the stimulatory effects of IL-1α

**Fig. 6:**
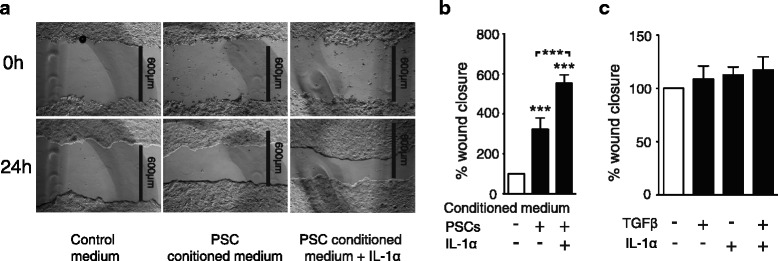
IL-1α induces PSC-stimulated migration of BxPC-3 cells. BxPC-3 cells were cultured in colonies to confluence and scratch wounds were established in the centre of the colony. The wound area was measured at 0 and 24 h and the data normalized to controls. **a**-**b** PSCs were cultured in serum free conditions for 3 days in the presence or absence of IL-1α (1 ng/ml) and the conditioned medium was transferred to the wound assays and incubated for 24 h. **c** Direct effects of TGFβ and IL-1α on BxPC-3 migration were analyzed by incubating BxPC-3 scratch wounds in the presence of IL-1α (1 ng/ml) and TGFβ (2 ng/ml) as single agents and in combination. Error bars represent S.E.M.; *p < 0.05, **p < 0.005, ***p < 0.001

We next investigated effects of PSCs on PDAC cells. Previous studies have demonstrated increased migration of pancreatic cancer cells in the presence of PSCs [14]. To study the involvement of IL-1α and TGFβ and the effects exerted by PSCs on migratory activity of PDAC cells, we assessed the effects of conditioned medium from unstimulated and IL-1α-stimulated PSCs on migration of BxPC-3 cells in a wound closure model. The conditioned medium from PSCs significantly increased the migration of the carcinoma cells compared to control medium (Fig. 6a). Moreover, conditioned medium from IL-1α-stimulated PSCs further enhanced cancer cell migration compared to both control medium and conditioned medium from unstimulated PSCs (Fig. 6b). This was not due to direct effects of IL-1α or TGFβ, since wound closure assays on BxPC-3 cells stimulated with IL-1α and TGFβ as single agents or in combination showed no effects on migration of BxPC-3 cells (Fig. 6c).

**Fig. 7:**
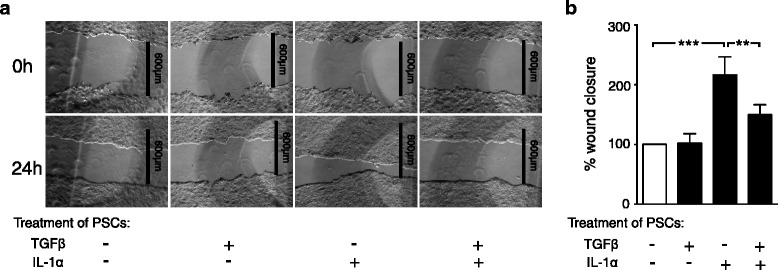
TGFβ inhibits cancer cell migration induced by IL-1α stimulated PSCs. **a**-**b** Conditioned medium harvested from PSCs cultured in the presence of IL-1α (1 ng/ml) and/or TGFβ (2 ng/ml) (3 days) was transferred to BxPC-3 wound assays. Wound recovery was determined after 24 h and the data normalized to unstimulated controls. Error bars represent S.E.M.; ***p < 0.001

We also investigated whether the stimulatory effects of IL-1α on the ability of PSCs to induce migration of BxPC-3 cells was affected by TGFβ. Medium from PSCs cultured in the presence of IL-1α and TGFβ, as single agents or in combination, was added to the BxPC-3 cells in the wound closure model. Conditioned medium from TGFβ-stimulated PSCs showed no effects on BxPC-3 migration compared to medium from unstimulated PSCs. On the other hand, conditioned medium from PSCs stimulated with TGFβ in combination with IL-1α significantly inhibited the migration of cancer cells compared to conditioned medium from IL-1α stimulated PSCs (Fig. 7a-b). Together this indicates an inhibitory role of TGFβ in PSC IL-1 signaling, affecting the potential of PSCs to induce cancer cell migration.

### Silencing of SMAD7 reduces the capacity of stellate cells to induce carcinoma cell migration

**Fig. 8:**
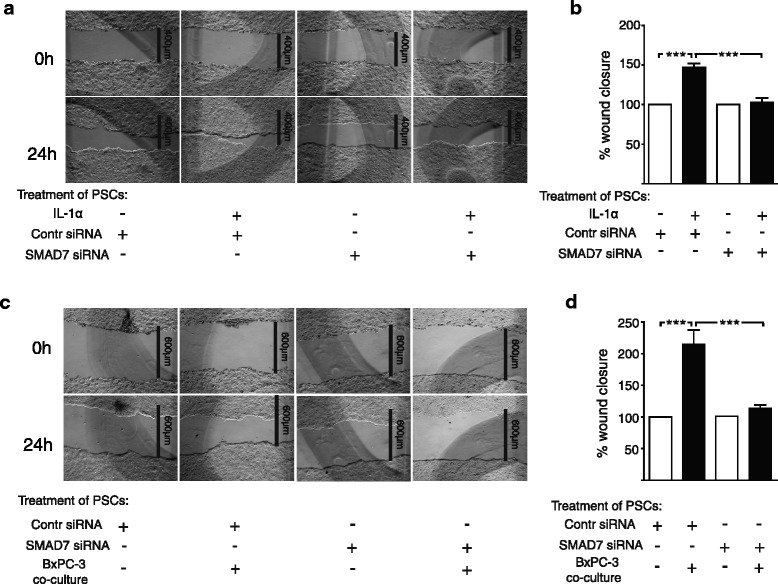
Inhibition of SMAD7 expression in PSCs reduces tumor stroma interactions and decrease cancer cell migration. **a**-**b** PSCs were transfected with non-targeting siRNA (control) or SMAD7 siRNA (100nM) for 3 days, followed by 2 days incubation in serum free medium and another 3 days of culture in the presence or absence of IL-1α (1 ng/ml) or (**c**-**d**) 12 h of indirectly co-culture with 5×10^5^ BxPC-3 cells and additionally 60 h of culture without any supplements. The supernatants were harvested 5 days post siRNA transfection and transferred to BxPC-3 scratch wound assays. Wound recovery was determined after 24 h and the data was normalized to controls. Error bars represent S.E.M.; ***p < 0.001

SMAD7-silenced PSCs were incubated in the presence or absence of IL-1α, and the effect of PSC-conditioned medium was assessed in a wound closure model using BxPC-3 pancreatic carcinoma cells. Silencing of SMAD7 reduced the ability of IL-1α to enhance the stimulating effect of PSC conditioned medium on the migration of the carcinoma cells compared to conditioned medium from non-silenced PSCs (Fig. 8a-b). In other experiments we co-cultured SMAD7-silenced PSCs with the IL-1α-positive BxPC-3 cell line and the conditioned medium was then added to the BxPC-3 wound closure model. The conditioned medium from the co-culture of BxPC-3 cells and SMAD7 silenced PSCs significantly inhibited the migration of carcinoma cells compared to the conditioned medium obtained from the co-culture of BxPC-3 and non-silenced PSCs (Fig. 8c-d). This strongly suggests that carcinoma cell migration is induced by IL-1α activation of PSCs, which can be inhibited by SMAD7 depletion. Together the data indicates a suppressive role for the TGFβ/SMAD signaling pathway in tumor stroma interactions.

## Discussion

In pancreatic adenocarcinoma the stroma forms the dominating bulk of the tumor tissue [42]. The pancreatic stellate cell is a key player in the complex regulation of the pancreatic tumor microenvironment [10]. IL-1α has previously been shown to activate PSCs and induce inflammatory responses in the tumor stroma [21]. TGFβ may act as both a tumor suppressor and a tumor promotor in pancreatic adenocarcinoma [43, 44]. In the present study we provide evidence for the involvement of TGFβ and SMAD in the regulation of IL-1 mediated responses in PSCs, focusing on inhibition of PSC induced migration of malignant cells.

In pancreatic adenocarcinomas, expression of TGFβ has been found to be enhanced in the cancer cells within the tumor mass, and overexpression of TGFβ was associated with poor survival [45]. In the tumor stroma, PSCs have also been demonstrated to produce TGFβ [46, 47]. We have previously reported that IL-1α produced by pancreatic cancer cells can activate and sustain the expression of inflammatory factors produced by PSCs [21] and promote cancer cell migration [48]. It has also been shown in pancreatic cancer cells that IL-1α exerts strong stimulatory effects on PSCs by inducing and sustaining a constitutive NFkB activation, associated with a metastatic phenotype [49, 50]. In the present study, we have demonstrated an inhibitory role of TGFβ in the regulation of IL-1α signaling between cancer cells and pancreatic stellate cells, with subsequent inhibition of the ability of PSCs to enhance cancer cell migration. We found that TGFβ reduced the expression of IL-1R1 on PSCs which subsequently diminished their ability to stimulate cancer cell migration upon IL-1α stimulation. The observation that TGFβ down-regulates the expression of IL-1R1on PSCs has to our knowledge not been reported previously. However, TGFβ has been found to reduce surface receptor expression of IL-1R1 in hematopoietic cells and T-cells, leading to reduced potential of the cells to respond to IL-1 stimulation [51].

The inhibitory effects of TGFβ on PSC expression of IL-1R1 was inhibited by blocking SMAD3, suggesting involvement of the canonical, SMAD-dependent TGFβ signaling pathway in the regulation of the IL-1R1 expression in these cells. SMAD7, an endogenous inhibitor of SMAD signaling, has been found to be overexpressed in pancreatic adenocarcinoma cells compared to healthy pancreatic tissue [52], but its expression and role in human PSCs is not known. We found a positive correlation between endogenous, unstimulated SMAD7 levels and the expression of IL-1R1 in PSCs (Fig. 4a). However, treatment of PSCs with TGFβ increased SMAD7 beyond the basal, endogenous level concomitant with a decrease in IL-1R1 (Fig. 4b). Notably, despite high levels of stimulated SMAD7, there was no increase in IL-1R1, which could be explained by negative feedback mechanisms attenuating the TGFβ signal. Silencing of endogenous SMAD7 reduced PSC expression of IL-1R1 and attenuated the effects exerted by IL-1α on the ability of PSCs to stimulate cancer cell migration. These results indicate that endogenous expression of SMAD7, by its ability to reduce the effect of TGFβ, is implicated in modulation of IL-1 signaling in pancreatic stellate cells.

SMAD7 might be a target for inhibition of IL-1α-dependent stimulation of pancreatic cancer cell migration. Interestingly, in inflammatory bowel disease, silencing of SMAD7 with anti-sense oligonucleotide treatment restored SMAD3 activation and reduced synthesis of inflammatory cytokines by endogenous TGFβ [53]. Our data showing a relationship between SMAD7 and IL-1R1 expression in PSCs is also consistent with recent data demonstrating decreased inflammation and clinical benefits for patients with active Crohn’s disease after blocking SMAD7 by a SMAD7 anti-sense oligonucleotide [54]. On the other hand, a previous report by Lee et al. has suggested an anti-inflammatory role of SMAD7 (and SMAD6) upon TGFβ treatment, through binding to Pellino-1, an IRAK1 adaptor protein which inhibits NFkB transcriptional activity and subsequently causes reduced expression of pro-inflammatory genes [55]. However, the TGFβ level required to down-regulate IL-1R1 is low compared to the levels required for upregulating the expression of SMAD7, indicating that different mechanisms are involved in the regulation of IL-1R1 activity and NFkB activity. This is further supported by several studies suggesting that Pellino 1 is dispensable for IL-1R1 signaling [56].

Activation of PSCs by IL-1 is associated with a specific inflammatory gene profile including chemokines, several inflammatory cytokines and some growth factors [21, 57]. Together, these factors support tumor progression in vivo and in vitro by enhancing tumor angiogenesis and cancer cell proliferation and migration [48, 57]. IL-1α inhibition by IL-1RA or IL-1α-neutralizing antibodies reduced the inflammatory PSC profile and inhibited cancer cell migration [21, 48]. Our present findings are consistent with the notion of a suppressing role for TGFβ/SMAD signaling in the regulation of the inflammatory PSC profile induced by IL-1α, with subsequent reduced migration of the carcinoma cells. The complex cellular cross-talk within the tumor stroma allows for the involvement of multiple signaling pathways in the regulation of cancer cell migration. Recently, Oyanagi et al. demonstrated in a 3D migration model that fibroblast-derived HGF level was reduced by addition of TGFβ, with concomitant inhibition of migration of pancreatic cancer cells, suggesting a direct inhibitory effect of TGFβ [58]. In our migration model no effect on cancer cell migration was observed after incubating PSCs in the presence of TGFβ alone, suggesting that other factors must be involved for TGFβ to exert its inhibitory effect on cancer cell migration. Furthermore, the existence of a vast cellular heterogeneity in the stromal compartment [59] might indicate diverse effects of TGFβ within the same tumor.

While much evidence has suggested a role of the tumor stroma as a supporter of pancreatic cancer progression, therapeutic stroma-directed strategies have so far not been successful [9], and recent experimental studies have actually raised doubt about the whole concept [60]. Depletion of pancreatic carcinoma-associated fibroblasts in genetically engineered mice has been found to accelerate disease progression [15, 16]. Taken together, the conflicting data [9, 12–16] indicate a more complex situation than has hitherto been recognized and which should be investigated in even more detail. More studies are needed to explore further the properties of pancreatic stellate cells, their interaction with the carcinoma cells, and the effects of the factors that regulate these cells.

## Conclusion

In the current work, we explored the role of the TGFβ-SMAD3 signaling pathway in pancreatic stellate cells and the consequences for tumor-stroma interactions and pancreatic adenocarcinoma cell migratory activity. We found that TGFβ down-regulated the expression of IL-1R1 on the stellate cells, which resulted in inhibition of the responses to IL-1α, with loss of the stimulatory effect of IL-1α on the ability of pancreatic stellate cells to enhance migration of the pancreatic carcinoma cells. Furthermore, depletion of SMAD7 upregulated the effects of TGFβ and reduced the expression of IL-1R1, leading to reduced stellate cell enhancement of carcinoma cell migration. There is a need to critically reassess the complexity of cross-talk between malignant and non-malignant cells of the pancreatic tumor. It might be of interest to explore SMAD7 in the stellate cells as a potential therapeutic target.

## Abbreviations

ECM, is short for extracellular matrix; EMT, is short for Epithelial-mesenchymal transition; IL-1R1, is short for Interleukin 1 receptor 1; IL-1α, is short for Interleukin 1α; IL-6, is short for Interleukin 6; IL-8, is short for Interleukin 8, IL-1RA, is short for Interleukin 1 receptor antagonist; PDAC, is short for pancreatic ductal adenocarcinoma; PDGF, is short for Platelet-derived growth factor; PSCs, is short for pancreatic stellate cells; TGFβ, is short for Transforming growth factor beta
